# The Optimal Effective Concentration Combination (OPECC) as a Novel Method for Evaluating the Effects of Binary Application of Antibacterial Compounds

**DOI:** 10.3390/microorganisms11040830

**Published:** 2023-03-24

**Authors:** Karl-Anton Hiller, Verena Wenzl, Eva-Maria Forster, Fabian Cieplik, Tim Maisch

**Affiliations:** 1 Department of Conservative Dentistry and Periodontology, University Hospital Regensburg, 93053 Regensburg, Germany; karl-anton.hiller@ukr.de (K.-A.H.);; 2 Department of Dermatology, University Hospital Regensburg, 93053 Regensburg, Germany; tim.maisch@ukr.de

**Keywords:** optimal effective concentration combination (OPECC), synergy, bacterial resistance, checkerboard assay, binary, combination therapy, antibiotics, antiseptics, *Escherichia coli*

## Abstract

Combination therapies appear to be beneficial for preventing bacterial resistance to antibacterial approaches. The aim of this study was to define and determine an optimal effective concentration combination (OPECC) for binary application of antibacterial compounds. The antiseptics chlorhexidine (CHX), benzalkonium chloride (BAC), and cetylpyridinium chloride (CPC), as well as the antibiotic ciprofloxacin (CIP), were tested against planktonic *Escherichia coli* in binary combinations by applying a checkerboard assay, and then evaluated according to the established synergism principles. Extending the checkerboard method, the optical density (OD) of the wells was measured photometrically. On the borderline between effective (OD = 0) and non-effective (OD > 0) eradication of the bacterial cultures, the OPECC was determined. Binary combinations of CPC or CHX with BAC were assessed as either synergistic or indifferent, respectively, while there was no OPECC to calculate. For all other binary combinations, an OPECC was derivable, and these were assessed as either synergistic or indifferent. In conclusion, the evaluation of the binary combination application of antibacterial compounds based on the checkerboard method was refined to such an extent that it was possible to determine at least one concentration pair that could be defined and considered as an OPECC, independently of the evaluation of the system according to the different synergy principles. In general, the method presented herein for determining an OPECC can be applied to any conceivable method or system aimed at the eradication of a pathogen.

## 1. Introduction

Even today, microbial infections play a major role in medicine, mostly due to the development of multiple-drug resistance [1,2]. This is even more alarming as the further development of antibacterial compounds is stagnating [3], and the development of antimicrobial resistance (AMR) might be causative of the death of 10 million people per year by 2050 [4,5]. In 2019, 4.95 million deaths were associated with AMR, and of these, 1.27 million deaths were caused by AMR. There is a need for forward-looking strategies to slow the spread of AMR [5]. Therefore, testing methods including, for example, combinations of antimicrobial compounds are important to enable new treatment strategies against infections caused by bacteria that are resistant to current therapy regimens [6,7]. The question of whether combination therapies are advantageous over monotherapies has not been fully clarified, and is the subject of much current research [8]. However, there is evidence that combination therapies might be advantageous, as it can be assumed that the simultaneous development of resistance is rather unlikely [9,10]. The binary effect of two antibacterial compounds is usually evaluated based on the principle of synergism. According to the American Society of Microbiology (ASM) [11], only a few methods are suitable for detecting synergistic effects of the binary application of two antibacterial compounds. The checkerboard test may be used to determine the binary interaction between two compounds [12,13]. For this test, two compounds are serially diluted and displayed two-dimensionally on a microtitration plate [13]. The checkerboard method is designed to produce wells with different concentrations of the single antimicrobials that provide information on the respective minimum inhibitory concentrations (MIC), as well as combination pairs of the antimicrobials used at different concentrations to determine the fractional inhibitory concentration (FIC) [11]. Based on this one-dimensional FIC for one compound, the combination of two compounds was proposed to be evaluated by the ∑FIC-Index [7,11,14]. Thereby, the ∑FIC is defined as “*…the concentration of each antibiotic in the combination that produces the specified effect is expressed as a fraction of the concentration that produces the same effect when the antibiotic is used alone, i.e., its fractional inhibitory concentration.*” [11]. Notably, different authors and scientific societies interprete the ∑FIC-Index differently. For example, ∑FIC = 1 is interpreted as *additivity* by Berenbaum and Dawis et al. [7,14], whereas the ASM [11] defines this as *indifferent* (Table 1).

The fluoroquinolone antibiotic ciprofloxacin (CIP) acts by inhibiting bacterial DNA gyrase, and is used in a wide range of clinical applications [15]. Chlorhexidine digluconate (CHX), benzalkonium chloride (BAC), and cetylpyridinium chloride (CPC) are cationic antiseptics that are commonly used in clinics as well as in over-the-counter products [16,17,18,19,20]. However, only a few studies have investigated the effect of combined application of antiseptics so far. Pons et al. evaluated the combination effect of CHX, BAC, and alkyltrimethylammonium bromide (cetrimide) against *Escherichia coli* and *Staphylococcus aureus.* The interactions between the combination pairs were described as synergistic and additive [21].

The synergism principle for evaluating binary combinations of antibacterial compounds does not specify an explicit combination of concentrations that will produce a result. However, it is based on the use of concentrations that effectively eliminate bacterial cultures [7,11,14].

The aim of the study was (1) to present definitions as to when concentration conditions are termed *effective* or *optimal*, and, based on these definitions, (2) to determine an *optimal effective concentration combination* (OPECC) of binary application of antimicrobial compounds against planktonic bacterial cultures.

## 2. Materials and Methods

### 2.1. E.coli Strains and Growth Conditions

*Escherichia coli* (ATCC 25922) was obtained from the American Type Culture Collection (Manassas, VA, USA). *E. coli* was cultured and stored on Mueller–Hinton agar plates (MH; provided by the Institute of Clinical Microbiology and Hygiene, University Hospital Regensburg, Germany, for this, 38.0 g of powdered Mueller–Hinton agar, ready to use (Merck KGaA, Darmstadt, Germany; composition: agar (17.0 g/L), beef infusion substances (2.0 g/L), casein hydrolysate (17.5 g/L), and starch (1.5 g/L)) were dissolved in 1 L Millipore water). A single colony of *E. coli* was picked from the agar plate and transferred to 5 mL media for an overnight (o/n) culture on an orbital shaker at 37 °C (180 rpm), with vigorous agitation to ensure proper aeration. Thereafter, the o/n planktonic cultures were centrifuged, the supernatant medium was removed, and pellets were dissolved in ion phosphate buffered saline (PBS; Dulbecco’s Phosphate Buffered Saline; Sigma-Aldrich, St. Louis, MO, USA). The bacterial suspension was adjusted to an optical density (OD) of 0.6, as measured at 600 nm (SPECORD 50 Plus, Analytik Jena, Jena, Germany).

### 2.2. Antibacterial Compounds

The antibacterial compounds benzalkonium chloride (BAC), chlorhexidine (CHX), cetylpyridinium chloride (CPC), and ciprofloxacin (CIP) were purchased from Sigma Aldrich. Considering the methods presented by Botelho et al. [11] and Schramm et al. [20], stock solutions of the antimicrobials BAC and CPC were prepared in distilled water (aqua dest.) and adjusted to a concentration of 128 µg/mL. For CHX, a stock solution of 20% (w-v; 200,000 µg/mL) was used. For CIP, a stock solution with a concentration of 128 µg/mL was prepared using aqua dest. with a pH of 4.8. All compounds were filter-sterilized (Acrodisc^®^ Syringe Filters, Pall, Newquaw, UK) to a pore size of 0.2 µm. For the determination of a sublethal dose for subsequent combination experiments, serial dilutions were prepared. BAC and CPC were diluted in PBS (Sigma-Aldrich); CHX and CIP in aqua dest., according to the respective manufacturer’s instructions.

### 2.3. Determination of Sublethal Concentrations, Area under the Curve (AUC), and Classical Methods

To determine the sublethal concentrations, *E. coli* cultures were treated with varying concentrations of BAC, CHX, CPC, and CIP in 48-well plates containing 250 µL (control: 500 µL) of MH broth (21.0 g of powdered Mueller–Hinton broth, ready to use (Merck; composition: beef infusion substances (2.0 g/L), casein hydrolysate (17.5 g/L), and starch (1.5 g/L))), which was dissolved in 1 L Millipore water. Compounds were added to varying final concentrations (µg/mL) for BAC (64-32-16-8-4-2-1), CPC (64-32-16-8-4-2-1-0.5), CIP (64-8-0.5-0.03-0.008-0.004-0.002-0.001), and CHX (50-12.5-3-1.6-0.8-0.4-0.1). Four wells were used per each concentration. Finally, 50 µL of the *E. coli* suspension was added. *E. coli* without any compound (positive control, four wells) and the respective compounds alone (two wells) served as controls. MH broth with the compounds alone present at different concentrations (blank values) was considered as an internal control, tested in duplicate, and used to correct the optical density (OD) values. The optical density was determined spectroscopically (VarioSkan Flash; SkanIt v. 2.4.5, Thermo Fisher Scientific, Vantaa, Finland) at 600 nm every 30 min from 0 to 180 min after treatment. In between, the plates were incubated at 37 °C under aerobic conditions without shaking, as previously established by our group [20]. Three independent experiments were performed for BAC, CPC, and CHX, and four for CIP.

**Figure 1:**
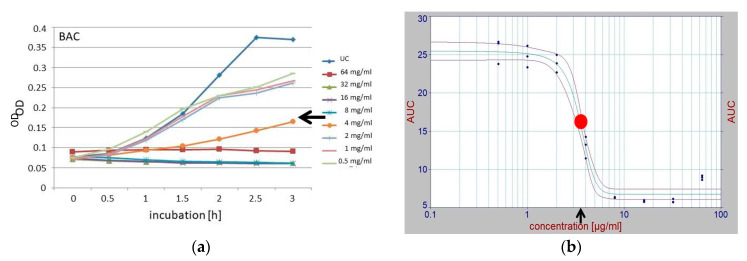
Determination of sublethal concentrations of the single compounds used for further combination experiments, as derived using the classical empirical method (**a**) and the newly introduced *area under the curve* (AUC) method (**b**), shown with the example of BAC. Various concentrations, from 0 to 128 mg/µL, were used to generate growth curves (as determined by optical density (OD) readings) during the 3 h of incubation, and were plotted in different colors (**a**). The area under the curve (AUC) values from each curve, representing the different concentrations of the applied antibacterial compound of panel (**a**), were calculated, visualized, and plotted against the corresponding concentrations, then fitted 2-dimensionally (TableCurve 2D); then, the inflection point (red dot) was derived (**b**) by applying the default modes of the utilized software. The concentration component of the inflection point was chosen as the sublethal concentration (**b**). Here, the sublethal concentrations derived empirically and by using the AUC method were 4 and 2.5 µg/mL (black arrows), respectively.

To specifically determine the sublethal concentrations for further use in the combination experiments, the areas under the growth curves (AUC) for each compound and concentration were calculated, and for each compound, the plot of these areas against their various concentrations was subjected to a 2-dimensional fit (TableCurve 2D, v 5.01; SYSTAT Software, Palo Alto, CA, USA). The concentration component of the inflection point derived from this fit was used as the sublethal concentration (Figure 1), and served as the initial concentration for the further combination experiments which applied the checkerboard method. This method was denominated as the AUC method. In parallel with this newly introduced AUC method, sublethal concentrations were determined classically from growth curves by visual assessment (Figure 1).

### 2.4. Combination Experiments

The checkerboard assay on 48-well plates was used for the combination experiments [11,13]. All six combination pairs of BAC, CPC, CHX, and CIP were examined. The dilution series were based on the calculated sublethal concentrations of the individual compounds, as derived by applying the AUC method (Table 2). The following final concentrations (µg/mL) were used: BAC 20-5-2.5-1.25-0.62; CIP 4.48-0.56-0.07-0.035- 0.018-0.009-0.002; CPC 24-12-6-3-1.5; and CHX 50-12.5-3-1.6-0.8-0.4-0.1. First, 300 µL MH broth was pipetted into each well, except the wells for single concentrations, where 450 µL MH broth was added. This was followed by adding 150 µL of the compounds and, finally, 50 µL *E. coli* suspension, to each well. Optical density was determined spectroscopically (VarioSkan Flash, SkanIt v. 2.4.5) at 600 nm both immediately (baseline, BL) and after incubation at 37 °C under aerobic conditions for 180 min. The plates were further incubated overnight at 37 °C under aerobic conditions. Three independent experiments were performed on the six respective combination pairs.

### 2.5. Synergism, Indifference, or Antagonism of the Binary Combinations

The well plates that were cultured overnight were classically analyzed for synergy by visual inspection. Hereby, it was decided whether a well was turbid or not. Based on these results, the synergism, indifference, and antagonism of the binary combinations of antibacterial compounds were evaluated according to the method described by Botelho [11].

### 2.6. Effective Concentrations and the Optimal Effective Concentration Combination (OPECC)

Generally, a certain condition applied in an antibacterial approach is defined to be *effective* if its use results in at least 99.9% (3 log_10_ steps) inactivation of the bacterial culture, applying the definition of an *antibacterial effect* used by the *American Society of Microbiology* [22,23]. In the present case, this means that a certain single concentration or binary combination of concentrations of the utilized compounds is effective if it eradicates the cultures in this sense.

**Figure 2:**
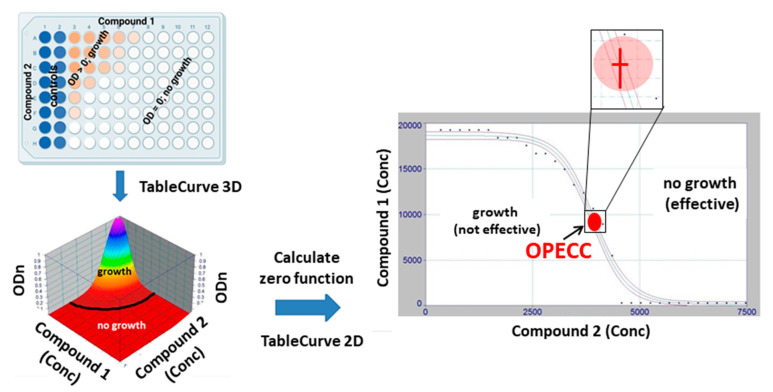
OPECC (*Optimal Effective Concentration Combination*) calculation. From a checkerboard plate (**top left panel**), the normalized OD values (ODn; *z*-axis, **bottom left panel**; ODn = OD values from plates incubated for 180 min, normalized to their baseline (0 min) values) were plotted against the respective concentration combination pairs (*x-y* plane) and fitted three-dimensionally (TableCurve 3D), with the result being *z = f (x,y)* (**bottom left panel**; colored 2 dimensional surface in 3 dimensional space). ODn = 0 is equivalent to no growth, thus indicating effective concentration combinations, whereas ODn > 0 is equivalent to growth, and, thus, non-effective concentration combinations. The set of 2-dimensional intersection points of this function *f* with the *x-y* plane was determined by applying the condition *z = f (x,y) =* 0 (black curve in **panel bottom left** separating no growth (effective concentration combinations) and growth (non-effective concentration combinations)). The points of this solution set in the 2-dimensional *x-y* (concentration) space were fitted 2 dimensionally (TableCurve 2D) and displayed together with their corresponding 95% confidence intervals (**right panel**). The inflection point (red dot) and the corresponding 95% confidence limits in both dimensions (insert) were determined (**right panel**) and used as describing parameters for the presentation of the results of the study. This inflection point was denominated as the *optimal effective concentration combination* (OPECC).

The method for determining the *Optimal Effective Concentration Combination* (OPECC) is detailed in Figure 2; the procedure was as follows. The optical density of each 180 min plate was normalized to its baseline value. For each binary combination of antibacterial compounds, the normalized optical density values of all plates were pooled together for further analysis. Embedded in the three-dimensional Euclidian *x-y-z*-space, the normalized OD values (ODn, *z*-axis) of a binary combination were plotted against the respective concentration combination pairs (*x-y* plane) and fitted three-dimensionally (TableCurve 3D, v 4.0; SYSTAT Software, Palo Alto, CA, USA), with the result being *z = f (x,y)*. The set of 2-dimensional intersection points of this function with the *x-y* plane was determined by applying the condition *z = f (x,y) =* 0. The points of this solution set in the 2-dimensional *x-y* (concentration) space were fitted 2-dimensionally (TableCurve 2D v 5.01; SYSTAT Software, Palo Alto, CA, USA) and displayed together with their corresponding 95% confidence intervals. The inflection point and the corresponding 95% confidence limits in both dimensions were determined and denominated as the *Optimal Effective Concentration Combination* (OPECC; Figure 2). This OPECC was used as a describing parameter for presentation of the results of the study.

## 3. Results

### 3.1. Sublethal Concentrations

For all tested compounds (BAC, CPC, CHX, and CIP), it was possible to calculate a point of inflection by applying the newly introduced *area under the curve* (AUC) procedure to derive sublethal concentrations (see Figure 1). These sublethal concentrations and those derived using the classical empirical procedures all fell into the same ranges (Table 2).

### 3.2. Optimal Effective Concentration Combination (OPECC)

For two of the six combination pairs (namely, CPC or CHX with BAC), an OPECC was not derivable, but for all other binary combinations, OPECCs could be determined. In these latter cases, each individual concentration component was lower than the concentration that would have needed to be used to eradicate the culture alone (Table 3).

### 3.3. Fractional Inhibitory Concentration (∑FIC) and Synergism

The results of the experiments leading to the fractional inhibitory concentration (∑FIC), as defined by Botelho [11], showed that the combinations BAC-CPC, CHX-CIP, and CHX -CPC were classified as synergistic, while all other binary combinations showed indifferent behavior (Table 4). Note that for each combination that was assessed to be synergistic based on the median of the ∑FIC, at least one of the samples was in the indifferent range, while for the indifferent combinations, the entire range of ∑FIC values fell into the indifferent range (Table 4).

### 3.4. Optimal Effective Concentration Combination (OPECC) and Synergism

For CPC or CHX with BAC, an OPECC was not derivable, and these combinations were assessed as synergistic or indifferent. For all other combinations with a derivable OPECC, two (CHX with CPC or CIP) combinations were synergistic, whereas the remaining two (CIP with BAC or CPC) were indifferent (Table 3 and Table 4).

## 4. Discussion

### 4.1. Compounds

In this study, the cationic antiseptics CHX, BAC, and CPC, which are commonly applied for disinfecting skin and mucous membranes, were used. For experimental reasons, the antibiotic CIP was also used. CIP belongs to the group of fluoroquinolones; it inhibits the enzyme gyrase and, thus, induces disruption of the DNA synthesis of bacteria [24,25]. This mechanism of action differs from that of the three antiseptics used. Normally, the lipid bilayer of bacteria is stabilized by cations, but if quaternary ammonium compounds (QACs) such as BAC and CPC, or bisguanides such as CHX, act on the bacteria, the cations are replaced by the positively charged head groups of the antibacterial compounds, which can bind to negatively charged phospholipids of the bacterial lipid bilayer [16,19]. Although all three antiseptics have the bacterial envelope as a common site of attack, they differ in detail. First, CHX has two positive charges, resulting in stronger bonding than BAC and CPC, which have a single positive charge. Furthermore, the hydrophobic regions of QACs become solubilized within the hydrophobic core of the cytoplasmic membrane, whereas those of CHX do not [26]. These minor differences in the mechanisms of action could explain the different results of the range-finding experiments. Both the large and the small differences in the antibacterial mode of action make all these compounds suitable model candidates to achieve the determination of an OPECC.

### 4.2. Sublethal Concentrations

The sublethal concentrations of the individual compounds were determined visually according to the established empirical method. An additional mathematical method was also used, namely, the newly introduced *AUC* (*area under the curve*) method. A sublethal concentration is defined as “*a dose or a concentration defined as inducing no statistically significant mortality in the experimental population*” [27]. The classical method allows for an individual influence of the evaluator assessing the turbidity of the well in binary decisions; therefore, it is necessary that this evaluation be performed only by experienced researchers. The AUC method does not allow an individual to have a subjective influence on the choice of the sublethal concentration. In our case, the empirically and mathematically derived AUC results were rather similar, as the senior author, a highly experienced microbiologist, applied the established method. Even the supposedly different BAC values of 2.5 and 4 µg/mL, which were derived by applying the AUC and the classical empirical methods, respectively, had no negative effect on the following combination experiments, since dilutions of the substances in both directions beyond the mentioned values were to be applied.

### 4.3. Minimum Inhibitory Concentration (MIC)

The MIC of CHX was reported to be 2 µg/mL when measured in planktonic *E. coli* cultures [28], similar to the 0.8 µg/mL found in our study. The MICs of BAC, CHX, and CPC, measured on *wet* and *dry* plates, were reported to be 16 to 64 µg/mL, 1 to 8 µg/mL, and 16 to 32 µg/mL, respectively, when tested against *E. coli* [29]. Although the latter study used the same laboratory strain as our study, the differences observed in our study may have been caused by the different culture conditions. From our laboratory, the MICs of BAC (10 µg/mL), CPC (10 µg/mL), and CHX (1 µg/mL) were reported for *E. coli* [30].

### 4.4. Incubation Period

For the evaluation of binary combination experiments, the colony-forming unit (CFU) method is often used, and the evaluation is frequently performed after 24 h [31,32]. Compared to this method, our study evaluated the results after the first 3 h of treatment as established and discussed in our laboratory [20] for the determination of the OPECC. In order to be able to relate our results to the established synergism method according to Botelho [11], the plates were subsequently additionally evaluated according to this method after 24 h.

### 4.5. Combination Experiments

Pons et al. tested the compounds CHX and BAC in binary combination against *E. coli*. The MICs for BAC were identical in both studies, but slightly different for CHX. In both studies, the system was evaluated as synergistic according to the Berenbaum [7] method [21].

### 4.6. Definition and Determination of the Optimal Effective Concentration Combination (OPECC)

An established method to evaluate binary combinations of antibacterial compounds is based on the checkerboard method [13]. The checkerboard method distinguishes between turbidity (no antibacterial effect; in our terms, *non-effective*) and non-turbidity (antibacterial effect; in our terms, *effective*) of the wells. The checkerboard method provides information about the MICs of single concentrations and about the effect of combination pairs of antimicrobials in different concentration combinations to determine fractional inhibitory concentrations. Based on these results, several definitions of *synergism, partial synergism, additivity, indifference*, and *antagonism* were presented by different authors [7,11,14], as shown in Table 1. All these definitions have one attribute in common: that they make a general statement about the utilized system without intending to provide guidance on how to calculate a synergistic, partially synergistic, indifferent, additive, or antagonistic combination of concentrations in the case of application.

**Figure 3:**
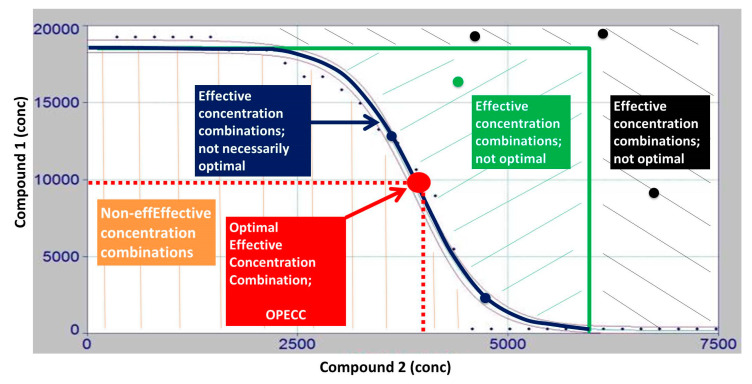
Discussion of the OPECC (*Optimal Effective Concentration Combination*) calculation process. The two-dimensional intersection line of the fitted three-dimensional curve (*z = f (x,y)*) with the *x-y* (concentration) plane (see Figure 2), i.e., the curve in the concentration plane resulting from the equation *z = f (x,y) =* 0 and here shown as a blue line, depicts the shift from non-turbid (z = ODn = 0, blue line; green and black areas, no growth, i.e., effective concentration combinations) to turbid (z = ODn > 0, brown area; growth, i.e., non-effective concentration combinations) wells (see Figure 2). Concentration pairs in the brown area show growth of the bacteria, and are, therefore, non-effective. For all concentration pairs in the green or black range (effective concentration pairs, exemplary dots in respective colors), both concentrations can be lowered down to the blue line, and the resulting, in both concentrations lower concentration pair, remains effective. Thus, the search for an optimal effective concentration pair is reduced to the blue line. On the blue line, it is true that a decrease in one of the two concentrations necessarily results in an increase in the other if the resulting concentration pair is to remain effective. The balance between the decrease in one concentration and the increase in the other is optimally achieved at the inflection point of the curve. Therefore, this inflection point is named the *optimal effective concentration combination* (OPECC).

**Figure 4:**
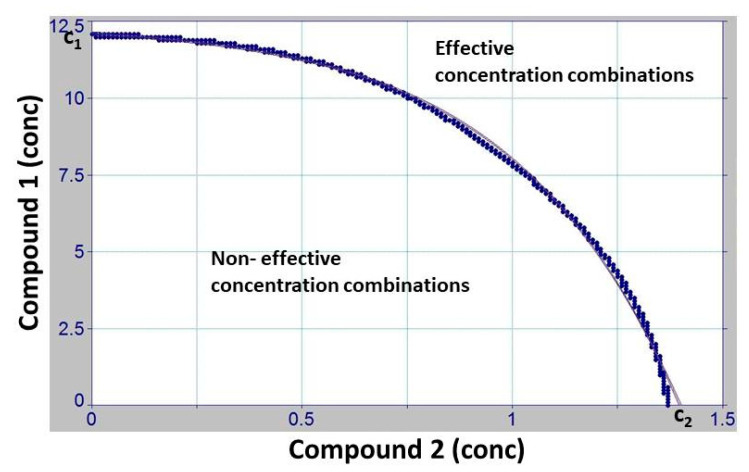
Discussion of the OPECC (*Optimal Point of Effective Concentration Combination*) calculation process. This is an example of a binary combination (CHX–BAC) for which no OPECC is directly determinable because this zero curve, representing the shift from non-effective to effective concentration pairs, is a strictly decreasing function without sign change of the derivation and, therefore, has no inflection point. In this case, the search for an optimal concentration pair is naturally still reduced to the blue line in Figure 3, shown here as a thin black line. If no further influencing constraints or factors are available, one of the single boundary concentration combinations ((C_2_; 0) or (0; C_1_)) can then be taken as the OPECC.

Extending the checkerboard method, the optical densities were measured; therefore, the dichotomous decisions (turbid or not turbid) for each well were refined to a continuous range, expressed as normalized optical densities ≥ 0. Thus, it was possible to visualize the results in a three-dimensional Euclidean space (Figure 2) and to distinguish between non-effective, effective, and optimal effective concentration combinations. A stepwise method was introduced to first find the effective, and then the optimal, combinations among these effective concentrations (Figure 2 and Figure 3). The latter were termed *optimal effective concentration combinations* (OPECCs). For a detailed discussion of why OPECCs should be considered optimal and effective, see the legend to Figure 3. Thus, an action instruction was given as to which particular combinations of concentrations should be optimally used to eradicate the bacteria in the binary application. It should be noted that this definition of an OPECC is independent of and does not touch any of the differing definitions of synergism, partial synergism, additivity, indifference, or antagonism. It may well be that a system is evaluated as not synergistic, but an OPECC is still to be determined. On the other hand, it is not necessarily true that in the case of an existing OPECC, the system must be synergistic. Three out of six of the binary combination pairs of the present study indicated a synergistic system, according to the classical checkerboard evaluation. Four of the six systems were evaluated as indifferent [11], including the combination pair BAC–CIP; however, it was possible to identify an OPECC in this system. The binary system BAC–CPC was evaluated as a synergistic system [11], but an OPECC was not able to be derived here (Table 2 and Table 3; Figure 4).

This points to another characteristic feature of the OPECC. If the zero curve, representing the shift from non-effective to effective concentration pairs (black line in the bottom left panel of Figure 2; blue line in Figure 3; and thin black line in Figure 4) has no inflection point, an OPECC cannot be determined directly, according to our definition. In this case, the search for an optimal concentration pair is, naturally, still reduced to the blue line in Figure 3 or the thin black line in Figure 4. If no further influencing constraints or factors are available, one of the single boundary concentration pairs (Figure 4) can then be taken as the OPECC. However, if other influencing factors are available, they can be considered to determine one of these combinations as optimally effective. Among such factors can be, for example, the price of a compound or the incubation period, or it could be considered that a compound can only be used up to a certain maximum concentration.

More generally, such and many other constraints can, but do not necessarily need to, be taken into account when determining the OPECC. It should be noted that if an OPECC exists, no other information needs to be available to determine an optimal effective concentration combination. If an OPECC exists in the present case, the interaction of the two compounds brings a positive effect such that less of each compound needs to be applied than when used alone.

### 4.7. Predictive Statements and Future Perspectives

The procedure for determining an OPECC based on the organism and the substances used can be extended to any pathogen, including bacteria, fungi, and parasites, as well as to clinical isolates and combinations thereof, etc. Theoretically, there are no restrictions on substances either. Aside from classical antibiotics and antiseptics, all conceivable procedures can be used, such as antimicrobial photodynamic therapy (aPDT), cold atmospheric plasma (CAP), etc. Generally, any procedure aimed at the eradication of any pathogen in the broadest sense can be applied. The only condition that must be met is that there must be a unique zero line in the three-dimensional space (Figure 2, bottom left panel) which can then be further analyzed (Figure 2, Figure 3 and Figure 4).

If the zero curve (Figure 2, panel right) has an inflection point, the binary application of the OPECC concentrations determined from it leads to reductions in both concentrations for both substances compared to single applications. This can have beneficial effects on the cost of treatment. Even more importantly, reducing concentrations can decrease the likelihood of side effects, toxicity, resistance development, etc.

## 5. Conclusions

In this study, for the first time, a method for determining sublethal concentrations that is free of subjective influences was introduced. The evaluation of the binary combination application of antibacterial agents based on the checkerboard method was refined to such an extent that it was possible to determine at least one concentration pair that could be defined and considered as an optimal effective concentration combination (OPECC), independently of evaluation of the system according to different synergy principles. In general, the method presented herein for determining an OPECC can be applied to any conceivable method or system aimed at the eradication of any pathogen.

## Figures and Tables

**Table 1:** Assessment of synergism, partial synergism, indifference, additivity, and antagonism based on the ∑FIC *-Index by different authors.

Berenbaum [7]	Botelho, The American Society of Microbiology [11]	Dawis et al. [14]
∑FIC < 1: synergism ∑FIC = 1: additivity ∑FIC > 1: antagonism	∑FIC ≤ 0.5: synergism 0.5 < ∑FIC ≤ 4.0: indifference ∑FIC > 4.0: antagonism	∑FIC ≤ 0.5: synergism 0.5 < ∑FIC < 1: partial synergism ∑FIC = 1: additivity 1 < ∑FIC < 4: indifference ∑FIC ≥ 4: antagonism
	
	

* “…*the concentration of each antibiotic in the combination that produces the specified effect is expressed as a fraction of the concentration that produces the same effect when the antibiotic is used alone, i.e., its fractional inhibitory concentration*” [11].

**Table 2:** Sublethal single concentrations derived and used for further combination experiments on all compounds tested, as determined by applying the newly introduced *area under the curve* (AUC) and classical empirical methods (Figure 1).

	Sublethal Concentration (µg/mL)	
Compound	AUC Method	Classical Empirical Method
BAC	2.5 (2.1; 2.8) *	4
CPC	6.1 (5.2; 7.0)	4
CHX	0.9 (0.8; 1.2)	0.8
CIP	0.035 (0.031; 0.058)	0.03

* 95% confidence limits in parentheses; see Figure 1b.

**Table 3:** OPECCs (*optimal effective concentration combinations*), including 95% confidence intervals, for binary combinations of the four tested compounds.

		OPECC ^#^ (µg/mL)			
		Compound 1		Compound 2	
Compound 1	Compound 2	Concentration	95% Conf ^§^	Concentration	95% Conf
CPC	BAC	−9 *		−9	
CHX	BAC	−9		−9	
CHX	CPC	1.11	1.10; 1.11	2.19	1.96; 2.42
CHX	CIP	0.93	0.92; 0.935	0.108	0.0105; 0.111
CIP	BAC	0.069	0.068; 0.0699	2.2633	2.214; 2.313
CIP	CPC	0.018	0.017; 0.0197	20.4	19.2; 21.5

**^#^** OPECCs were derived according to the procedure described in Figure 2 and discussed in Figure 3. Normalized optical density readings of checkerboard plates were three-dimensionally fitted (TableCurve 3D). The intersection of this fit with the concentration plane (interface between effective and non-effective eradication) was fitted two-dimensionally (TableCurve 2D). The concentration pair, including 95% confidence limits in both directions at the point of inflection of this curve, was derived and called the *optimal effective concentration combination* (OPECC) (see insert in Figure 2). ^§^ 95% confidence limit. * −9 indicates that no point of inflection was derivable, in this case use one of the single boundary concentrations (see Figure 4).

**Table 4:** Fractional inhibitory concentration (ƩFIC) presented as median and range for binary combinations of the four tested compounds.

		ƩFIC *				
Compound 1	Compound 2	Median	Range	Assessment	Sample Size	OPECC Exists ^§^
CPC	BAC	0.31	0.19–0.56	synergism	3	no
CHX	BAC	0.66	0.52–0.75	indifference	3	no
CHX	CPC	0.50	0.39–0.52	synergism	3	yes
CHX	CIP	0.50	0.18–0.78	synergism	3	yes
CIP	BAC	0.63	0.38–0.75	indifference	3	yes
CIP	CPC	1.00	1.00–1.00	indifference	1	yes

* “…*the concentration of each antibiotic in the combination that produces the specified effect is expressed as a fraction of the concentration that produces the same effect when the antibiotic is used alone, i.e., its fractional inhibitory concentration*” [12]. ^§^ See Table 3. ƩFIC was calculated from experiments with the indicated sample sizes, as defined by Botelho [11], for the six binary combinations of antibacterial compounds tested against *E. coli*. Binary combinations were assessed as synergistic or indifferent, and the coarsened information from Table 3 on the existence of an optimal effective concentration combination (OPECC) was also used.

## Data Availability

The data presented in this study are available upon request from the corresponding author.

## References

[1] Barra F., Roscetto E., Soriano A.A., Vollaro A., Postiglione I., Pierantoni G.M., Palumbo G., Catania M.R. (2015). Photodynamic and Antibiotic Therapy in Combination to Fight Biofilms and Resistant Surface Bacterial Infections. Int. J. Mol. Sci..

[2] Laxminarayan R. (2022). The overlooked pandemic of antimicrobial resistance. Lancet.

[3] Rossolini G.M., Mantengoli E. (2008). Antimicrobial resistance in Europe and its potential impact on empirical therapy. Clin. Microbiol. Infect..

[4] O’Neill J. (2016). Tackling Drug-Resistant Infections Globally: Final Report and Recommendations.

[5] Antimicrobial Resistance Collaborators (2022). Global burden of bacterial antimicrobial resistance in 2019: A systematic analysis. Lancet.

[6] Choudhary M.I., Römling U., Nadeem F., Bilal H.M., Zafar M., Jahan H., Ur-Rahman A. (2022). Innovative Strategies to Overcome Antimicrobial Resistance and Tolerance. Microorganisms.

[7] Berenbaum M.C. (1978). A method for testing for synergy with any number of agents. J. Infect. Dis..

[8] Schmid A., Wolfensberger A., Nemeth J., Schreiber P.W., Sax H., Kuster S.P. (2019). Monotherapy versus combination therapy for multidrug-resistant Gram-negative infections: Systematic review and meta-analysis. Sci. Rep..

[9] Mouton J.W. (1999). Combination therapy as a tool to prevent emergence of bacterial resistance. Infection.

[10] Wright G.D., Sutherland A.D. (2007). New strategies for combating multidrug-resistant bacteria. Trends Mol. Med..

[11] Botelho M.G. (2000). Fractional inhibitory concentration index of combinations of antibacterial agents against cariogenic organisms. J. Dent..

[12] Wozniak A., Grinholc M. (2018). Combined Antimicrobial Activity of Photodynamic Inactivation and Antimicrobials-State of the Art. Front. Microbiol..

[13] Jenkins S.G., Schuetz A.N. (2012). Current concepts in laboratory testing to guide antimicrobial therapy. Mayo Clin. Proc..

[14] Dawis M.A., Isenberg H.D., France K.A., Jenkins S.G. (2003). In vitro activity of gatifloxacin alone and in combination with cefepime, meropenem, piperacillin and gentamicin against multidrug-resistant organisms. J. Antimicrob. Chemother..

[15] Zhang G.-F., Liu X., Zhang S., Pan B., Liu M.-L. (2018). Ciprofloxacin derivatives and their antibacterial activities. Eur. J. Med. Chem..

[16] Cieplik F., Jakubovics N.S., Buchalla W., Maisch T., Hellwig E., Al-Ahmad A. (2019). Resistance toward Chlorhexidine in Oral Bacteria—Is There Cause for Concern?. Front. Microbiol..

[17] Mao X., Auer D.L., Buchalla W., Hiller K.-A., Maisch T., Hellwig E., Al-Ahmad A., Cieplik F. (2020). Cetylpyridinium Chloride: Mechanism of Action, Antimicrobial Efficacy in Biofilms, and Potential Risks of Resistance. Antimicrob. Agents Chemother..

[18] Pereira B.M.P., Tagkopoulos I. (2019). Benzalkonium Chlorides: Uses, Regulatory Status, and Microbial Resistance. Appl. Environ. Microbiol..

[19] Muehler D., Sommer K., Wennige S., Hiller K.-A., Cieplik F., Maisch T., Späth A. (2017). Light-activated phenalen-1-one bactericides: Efficacy, toxicity and mechanism compared with benzalkonium chloride. Future Microbiol..

[20] Schramm S., Hiller K.-A., Cantzler S., Weilemann H., Cantzler M., Zimmermann J.L., Cieplik F., Maisch T. (2020). The Latest Time Point of Retreatment (LTPR) as a Novel Method to Determine Antibacterial Effects for Binary Use of Cold Atmospheric Plasma and Conventional Agents. Front. Microbiol..

[21] Pons J.L., Bonnaveiro N., Chevalier J., Crémieux A. (1992). Evaluation of antimicrobial interactions between chlorhexidine, quaternary ammonium compounds, preservatives and excipients. J. Appl. Bacteriol..

[22] Pearson R.D., Steigbigel R.T., Davis H.T., Chapman S.W. (1980). Method of reliable determination of minimal lethal antibiotic concentrations. Antimicrob. Agents Chemother..

[23] Taylor P.C., Schoenknecht F.D., Sherris J.C., Linner E.C. (1983). Determination of minimum bactericidal concentrations of oxacillin for Staphylococcus aureus: Influence and significance of technical factors. Antimicrob. Agents Chemother..

[24] Brun P., Bernabè G., Marchiori C., Scarpa M., Zuin M., Cavazzana R., Zaniol B., Martines E. (2018). Antibacterial efficacy and mechanisms of action of low power atmospheric pressure cold plasma: Membrane permeability, biofilm penetration and antimicrobial sensitization. J. Appl. Microbiol..

[25] Ball A.P. (1986). Overview of clinical experience with ciprofloxacin. Eur. J. Clin. Microbiol..

[26] Gilbert P., Moore L.E. (2005). Cationic antiseptics: Diversity of action under a common epithet. J. Appl. Microbiol..

[27] Desneux N., Decourtye A., Delpuech J.-M. (2007). The sublethal effects of pesticides on beneficial arthropods. Annu. Rev. Entomol..

[28] Thongrueang N., Liu S.-S., Hsu H.-Y., Lee H.-H. (2022). An in vitro comparison of antimicrobial efficacy and cytotoxicity between povidone-iodine and chlorhexidine for treating clinical endometritis in dairy cows. PLoS ONE.

[29] Roedel A., Dieckmann R., Makarewicz O., Hartung A., Noll M., Pletz M.W., Dahouk S.A., Vincze S. (2020). Evaluation of a Newly Developed Vacuum Dried Microtiter Plate for Rapid Biocide Susceptibility Testing of Clinical Enterococcus Faecium Isolates. Microorganisms.

[30] Schwarz S.R., Hirsch S., Hiergeist A., Kirschneck C., Muehler D., Hiller K.-A., Maisch T., Al-Ahmad A., Gessner A., Buchalla W. (2021). Limited antimicrobial efficacy of oral care antiseptics in microcosm biofilms and phenotypic adaptation of bacteria upon repeated exposure. Clin. Oral Investig..

[31] Ronqui M.R., de Coletti T.M.S.F.A., de Freitas L.M., Miranda E.T., Fontana C.R. (2016). Synergistic antimicrobial effect of photodynamic therapy and ciprofloxacin. J. Photochem. Photobiol. B.

[32] Iluz N., Maor Y., Keller N., Malik Z. (2018). The synergistic effect of PDT and oxacillin on clinical isolates of Staphylococcus aureus. Lasers Surg. Med..

